# Rare Pancreatic Arterial Variations: A Cadaveric Case Report

**DOI:** 10.7759/cureus.82446

**Published:** 2025-04-17

**Authors:** Isaiah J Pratt, Abigail L Ryan, Emily J Gresner, Angela R Loczi-Storm, Edith Sperling

**Affiliations:** 1 College of Medicine, College of Osteopathic Medicine of the Pacific-Northwest, Western University of Health Sciences, Lebanon, USA; 2 Anatomy, Western University of Health Sciences, Lebanon, USA; 3 Anatomical Sciences, Western University of Health Sciences, Lebanon, USA

**Keywords:** cadaver, dorsal pancreatic artery, pancreas, pancreatic blood supply, rare anatomical variant

## Abstract

The dorsal surfaces of the pancreatic head and body receive a significant amount of blood supply from the dorsal pancreatic artery, whose common origins include the splenic artery, superior mesenteric artery, celiac trunk, and the common hepatic artery, with the most common being the splenic artery. In the majority of cases, the dorsal pancreatic artery is present in some form. In this case study, the dorsal aspect of the head and body of the pancreas is supplied by a large artery not commonly found in the literature, referred to here as the right posterior pancreatic artery (RPPA). The RPPA was found during routine cadaveric dissection in a medical school anatomy lab. Arising from the gastroduodenal artery, it traveled along the dorsal aspect of the head and body of the pancreas and terminated at the abdominal aorta, replacing dorsal pancreatic, large pancreatic, or short pancreatic arteries. The RPPA’s location at the head of the pancreas, in conjunction with its size and a lack of additional blood flow from the dorsal, large, or short pancreatic arteries, is useful knowledge for surgical interventions involving the pancreas.

## Introduction

The pancreas is an intra-abdominal, secondarily retroperitoneal organ with both exocrine and endocrine functions [1,2]. The exocrine component secretes alkaline pancreatic juice into the duodenum via one or two pancreatic ducts to aid in digestion [1,2]. The endocrine component produces at least five hormones, which are transported via circulation to intended targets [2].

Pancreas embryogenesis begins in about the fifth week, with the duct developing first, followed by glandular lobules [3]. Two buds, dorsal and ventral, arise on opposite sides of the developing duodenum [2,3]. Between the fifth and tenth weeks of gestation, midgut rotation repositions the ventral bud posterior to the dorsal bud, and fusion occurs around the seventh week [2,3]. The embryological changes in gut anatomy lead to frequent anatomical variations [2]. The pancreas’ vasculature is determined largely by gut vasculature development, both vasculogenesis and angiogenesis [2]. The pancreas is typically supplied by branches of the celiac and superior mesenteric arteries, which develop from an arterial anastomosis arising from the aorta, and branches from the splenic artery [2,3]. The blood flow of the pancreas affects its exocrine and endocrine functions [2].

While the vascular supply of the pancreas is variable, the most frequently encountered arterial sources are the hepatic, splenic, and superior mesenteric arteries [1,2]. The head of the pancreas, which is surrounded on three sides by the duodenum, most often receives its blood supply from the gastroduodenal artery via the anterior and posterior pancreaticoduodenal arteries [1-5]. The body of the pancreas is typically supplied by the dorsal pancreatic artery, which arises from the splenic artery [1-6]. The dorsal pancreatic artery courses posterior to the pancreatic body and provides branches to the head, body, and tail, making it a critical vessel to consider during surgical procedures such as pancreatectomy or pancreaticoduodenectomy (Whipple procedure) [7-9].

Recently, the first approach of the artery has gained popularity in pancreatic resections. This technique prioritizes dissection and, when necessary, ligation of key arteries before tumor resection, reducing intraoperative blood loss and venous congestion [7,8,10]. Ligation of the dorsal pancreatic artery, in particular, has been associated with reduced blood loss, but its variable anatomy may complicate this step, underscoring the importance of identifying anatomical variants [7,8].

Although variations in the vascular supply of abdominal organs are common, it is important to report these variations for surgeons to be aware [6]. This case report documents a variation of pancreatic blood supply not commonly found in the literature, which was discovered during routine human cadaver dissection by medical students in the anatomy lab at the Western University of Health Sciences.

## Case presentation

**Figure 1:**
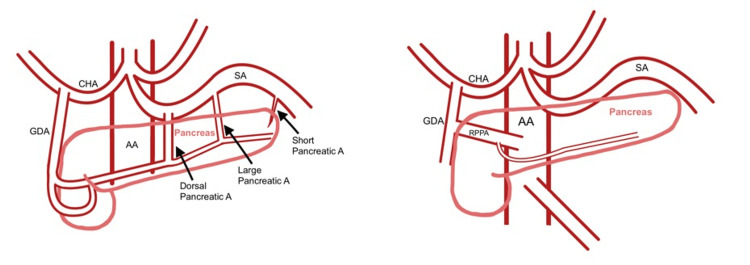
Typical anatomy (left) with the variation described here (right) AA: abdominal aorta, CHA: common hepatic artery, GDA: gastroduodenal artery, SA: splenic artery, RPPA: right posterior pancreatic artery (the variation)

This study was approved by the Institutional Review Board of the Western University of Health Sciences (#2128977) (Appendix 1). During the routine cadaveric dissection of a female donor in her 80s, an anomalous artery supplying the head and body of the pancreas was discovered. Measuring approximately 1 cm in diameter, this artery originated from the gastroduodenal artery inferior to the posterior superior pancreaticoduodenal artery and superior to the anterior superior pancreaticoduodenal artery. Here, we refer to it as the right posterior pancreatic artery (RPPA). Figure 1 depicts the normal anatomy (left) and atypical anatomy of the RPPA (right). 

**Figure 2:**
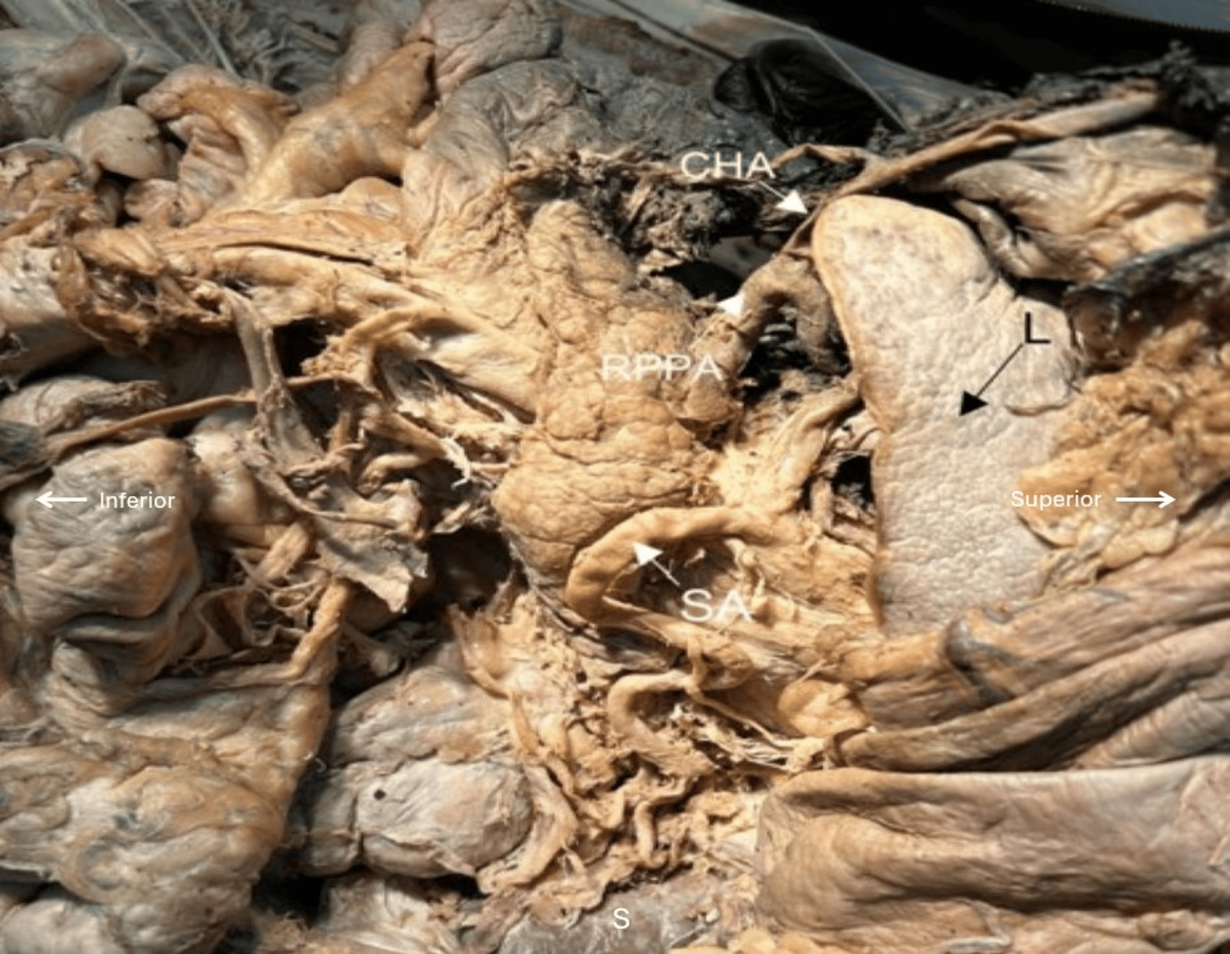
Celiac trunk with common hepatic artery (CHA) running toward the liver (L) with the right posterior pancreatic artery (RPPA) originating from the gastroduodenal artery. Splenic artery (SA) shown with no dorsal pancreatic, large pancreatic, or short pancreatic arteries. SA continues to the spleen (S)

**Figure 3:**
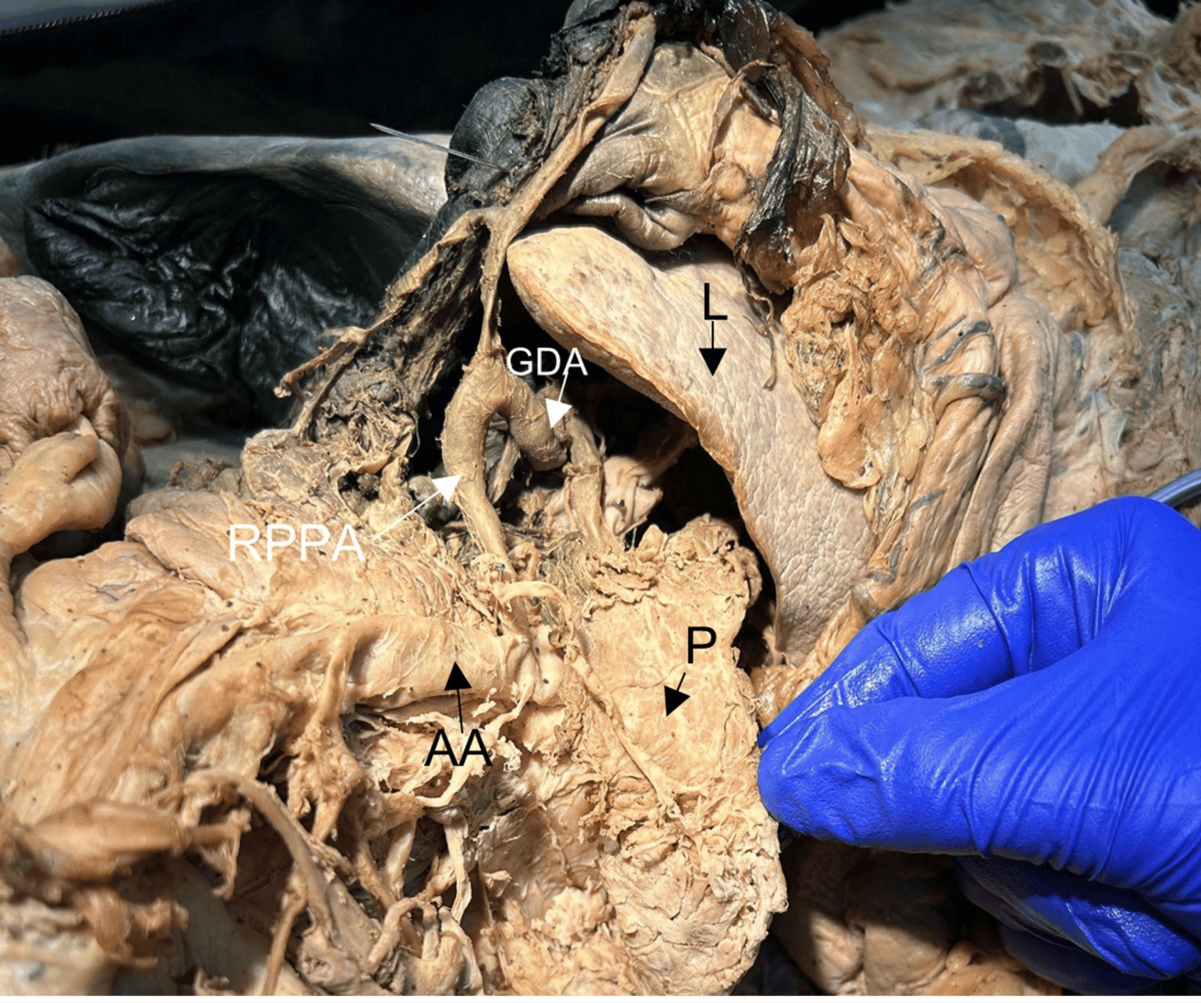
Reflected pancreas (P) showing the right posterior pancreatic artery (RPPA) arising from the gastroduodenal artery (GDA) and ending at the abdominal aorta (AA) in the absence of a dorsal pancreatic artery

The RPPA traveled medially and posteriorly to the body of the pancreas, ultimately connecting to the abdominal aorta between the celiac trunk and the superior mesenteric artery. Just before reaching the abdominal aorta, the RPPA gave off a branch that coursed leftward along the posterior-inferior surface of the pancreas, terminating mid-pancreas at the site where the splenic artery typically embeds (Figures 2, 3). 

Notably, the artery in this cadaver lacked the usual dorsal pancreatic, large pancreatic, and short pancreatic arteries, though the artery to the tail of the pancreas was present (Figure 2). Additionally, the superior mesenteric artery lacked the typical posterior-inferior and anterior-inferior pancreaticoduodenal arteries. However, the superior mesenteric artery did give off a small branch to the left, which ascended parallel to the abdominal aorta, posterior to the pancreas, and joined the abdominal aorta on the left lateral aspect at the same level at which the RPPA joined the abdominal aorta. All other branches of the celiac and superior mesenteric trunks appeared typical.

## Discussion

This case study presents the occurrence of a rare pancreatic arterial variant in which an artery running between the gastroduodenal artery and the aorta supplied the pancreatic head and body in the absence of a dorsal pancreatic, long pancreatic, or short pancreatic artery(ies). Instead, the blood supply to the body of the pancreas was coming from the artery we called the right posterior pancreatic artery (RPPA). This artery was not found in the literature describing pancreatic arterial supply variants [1-5]. More common pancreatic variations include a gastro-pancreatic-colic trunk (prevalence 0.61) and a gastro-pancreatic trunk (prevalence 0.34); there are variations within these variants as well [11].

In this case, an artery-first approach to a pancreatectomy or pancreaticoduodenectomy may indicate ligation of the anomalous RPPA as it arose from the gastroduodenal artery and aorta [7,8]. However, visualization would be difficult, particularly without knowing the course of the artery beforehand. A recent study on outcomes of pancreaticoduodenectomies recommended presurgical CT angiography to visualize the anatomy of the dorsal pancreatic artery and the presence and type of intrapancreatic arcades to reduce the complication of a postoperative pancreatic fistula [9]. Risk factors have been found to be able to be estimated using presurgical contrast-enhanced CT [9,12]. Studies support that collateral and intrapancreatic variations may affect postsurgical outcomes [9].

Pancreatic arterial variations are also important to know for pancreatic grafts, as studies indicate that splenic arterial dominance significantly improves transplantation success [13]. Oversights in organ procurement, due partly to anatomical variations, account for close to 20% of insufficient pancreatic allografts [14]. The dorsal pancreatic artery in particular can be accidentally cut, leading to impaired blood supply and poor revascularization in transplant surgeries [14]. Pancreatic transplantation dependent on one large artery, typically the splenic artery, can allow sufficient perfusion to the pancreas [13]. The pancreas in this case would likely have been a poor candidate for transplantation due to lack of splenic arterial supply.

## Conclusions

We report a rare arterial variant supplying the head and body of the pancreas, in which a branch of the gastroduodenal artery connects to the aorta between the celiac trunk and superior mesenteric artery, replacing the typical dorsal, long, and short pancreatic arteries. Case studies of anatomical variations in donor patients have proven valuable in raising awareness and enhancing surgical outcomes. Recognizing these variations can help minimize complications and inform presurgical planning. While pancreatic vascular variations are common, documenting specific cases remains essential for improving anatomical understanding and guiding operative approaches.

## Figures and Tables

**Figure 4:**
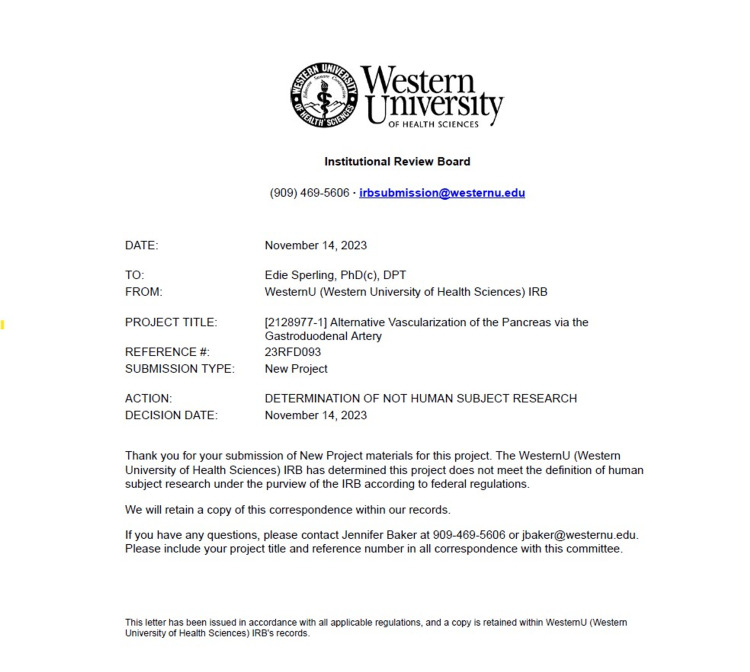
IRB approval letter
